# Improving knowledge on the management of diabetes mellitus during Ramadan

**DOI:** 10.1016/j.jtumed.2025.10.005

**Published:** 2025-11-19

**Authors:** A.A. Arie Widyastuti, Nurul H. Muchtar, Jerry Nasarudin, M. Ikhsan Mokoagow, Marina Epriliawati, Ika Saptarini, Ida A. Made Kshanti

**Affiliations:** a Internal Medicine Department, Fatmawati General Hospital, Jl. RS. Fatmawati Raya, Cilandak Bar., Kec. Cilandak, Kota Jakarta Selatan, Daerah Khusus Ibukota, Jakarta, Indonesia; b General Practitioner, Fatmawati General Hospital, Jl. RS. Fatmawati Raya, Cilandak Bar., Kec. Cilandak, Kota Jakarta Selatan, Daerah Khusus Ibukota, Jakarta, Indonesia; c Endocrine Diabetes & Metabolism, Internal Medicine Department, Fatmawati General Hospital, Jl. RS. Fatmawati Raya, Cilandak Bar., Kec. Cilandak, Kota Jakarta Selatan, Daerah Khusus Ibukota, Jakarta, Indonesia; d National Research and Innovation Agency Republic of Indonesia Bogor, Jalan Raya Jakarta-Bogor Km.46 Kec. Cibinong, Kabupaten Bogor, Jawa Barat, Indonesia

**Keywords:** Diabetes, Education, Fasting, Knowledge, Ramadan, داء السكري من النوع الثاني, رمضان, الصيام, التثقيف الصحي, المعرفة

## Abstract

**Introduction:**

Ramadan fasting is obligatory for adult Muslims but poses significant health risks for individuals with type 2 diabetes mellitus (T2DM), including hypoglycemia and hyperglycemia. This study evaluated baseline knowledge and the impact of a targeted educational program on DM management during Ramadan among adults with T2DM.

**Methods:**

A single-arm pre-test/post-test design was conducted at Fatmawati General Hospital (February–April 2023) in a consecutive sample of 116 Muslim patients with T2DM who were planning to fast. Participants received a Ramadan-focused education session (online or offline) covering attendance, dietary planning, risk stratification, and self-monitoring. Knowledge was assessed with a 24-item questionnaire (6 attendance-related, 18 content-related questions). Data were analyzed using IBM SPSS Statistics version 22, with Kolmogorov–Smirnov tests for normality and paired *t*-tests or Wilcoxon signed-rank tests for pre-post comparisons (α = 0.05).

**Results:**

In the pre-intervention assessment, 50.9% of participants attained a “good” overall knowledge score, which increased significantly to 90.5% following the Ramadan-focused education program (Wilcoxon signed-rank test, p = 0.001). Domain-specific analyses revealed marked gains in understanding of fasting risks (from 63.8% to 92.2%), complex carbohydrate principles (37.9%–63.8%), physical activity recommendations (69.0%–78.5%), medication-related risk awareness (58.6%–81.0%) and risk stratification methods (83.9%–92.0%). By contrast, knowledge of the appropriate glucose-monitoring frequency improved modestly, reaching 67.2% post-intervention.

**Conclusion:**

A structured, Ramadan-focused education program markedly enhances knowledge of safe fasting practices in patients with T2DM. To further optimize outcomes, future interventions should intensify focus on specific dietary strategies, sulfonylurea-related hypoglycaemia risks, and standardized glucose-monitoring protocols.

## الملخص

**أهداف البحث:**

يُعَدّ الصيام في شهر رمضان فريضة على المسلمين البالغين، إلا أنه قد يُعرِّض المصابين بداء السكري من النوع الثاني لمخاطر صحية مثل نقص أو ارتفاع سكر الدم. تهدف هذه الدراسة إلى تقييم مستوى المعرفة الأساسية لدى مرضى السكري من النوع الثاني، وقياس أثر برنامجٍ تثقيفيٍّ موجهٍ حول إدارة المرض أثناء الصيام في تحسين معارفهم المرتبطة بالصيام الآمن.

**طرق البحث:**

أُجريت دراسة شبه تجريبية بتصميم قبلي–بعدي في مستشفى فاطمة واتـي العام بإندونيسيا خلال الفترة من فبراير إلى أبريل 2023، *شملت 116 مريضً*ا مسلمًا مصابًا بداء السكري من النوع الثاني كانوا ينوون الصيام. تلقّى المشاركون جلسة تثقيفية متخصّصة حول الصيام في رمضان، تضمنت موضوعات عن الحضور والاستعداد، والتخطيط الغذائي، وتحديد درجة الخطورة، والمراقبة الذاتية لمستويات الجلوكوز. جرى تقييم المعرفة باستخدام استبانة مكوّنة من 24 سؤالاً، وحُللت الفروق بين النتائج قبل وبعد البرنامج باستخدام اختبار **ويلكوكسون** بدرجة دلالة (p<0.05).

**النتائج:**

أظهرت نتائج التقييم المبدئي أن 50.9% من المشاركين حققوا مستوى “جيدًا” من المعرفة العامة، وارتفعت هذه النسبة إلى 90.5% بعد تنفيذ البرنامج التثقيفي (p=0.001). كما تحسنت المعرفة في جميع المجالات الفرعية، لا سيما فهم مخاطر الصيام (من 63.8% إلى 92.2%)، ومبادئ تناول الكربوهيدرات المعقدة (من 37.9% إلى 63.8%)، والنشاط البدني الآمن (من 69.0% إلى 78.5%)، والإدراك المرتبط بمخاطر الأدوية (من 58.6% إلى 81.0%)، وطرق تصنيف الخطورة (من 83.9% إلى 92.0%). أما معرفة تكرار قياس سكر الدم فشهدت تحسنًا محدودًا (إلى 67.2%).

**الاستنتاجات:**

يُظهر البرنامج التثقيفي المركَّز على شهر رمضان فاعلية كبيرة في تعزيز معرفة المرضى بداء السكري من النوع الثاني حول ممارسات الصيام الآمن، مما يدعم أهمية دمج التثقيف الصحي الموجه ثقافيًا ودينيًا ضمن برامج الرعاية المزمنة. كما توصي الدراسة بزيادة التركيز على الجوانب الغذائية التفصيلية، ومخاطر نقص سكر الدم المرتبطة بعقاقير السلفونيل يوريا، ووضع بروتوكولات موحّدة للمراقبة الذاتية للجلوكوز خلال الصيام.

## Introduction

Ramadan is the ninth month of the Islamic lunar calendar, and fasting throughout its duration constitutes one of the Five Pillars of Islam for adult Muslims who have reached puberty. For 29–30 days each year, observers must refrain from ingesting any food or fluid from dawn (suhoor) until sunset (iftar). Certain groups are exempted from this obligation, including older adults, pregnant or lactating women, and individuals with acute or chronic illnesses such as diabetes mellitus (DM). Muslims who are unable to fast for valid reasons are obligated to perform Fidya, compensation for each missed day, typically by providing meals or making monetary donations to impoverished individuals.^1^, ^2^, ^3^ The burden of DM continues to rise globally, with an estimated 463 million adults affected in 2019 and projections indicating an increase to 700 million by 2045.^4^ In parallel, the Muslim population was estimated at approximately 1.9 billion individuals in 2019, accounting for nearly 24% of the world's population. Despite the potential risks associated with prolonged fasting, epidemiological studies indicate that most Muslims with T1DM or T2DM opt to observe the Ramadan fast, with the majority fasting for at least half of the month (i.e., 15 days or more).^5^

Fasting during Ramadan may precipitate acute glycemic disturbances, chiefly hypoglycemia and hyperglycemia, in patients with DM. The International Diabetes Federation–Diabetes and Ramadan (IDF-DAR) Practical Guidelines categorize individuals with diabetes into low-, moderate-, and high-risk categories for fasting. Patients in the high-risk category are generally advised against fasting; many nonetheless choose to observe the fast. Recognizing this, the IDF-DAR alliance has delineated specific prerequisites to ensure that such patients undertake fasting as safely as possible. Before Ramadan, patients must undergo a structured pre-Ramadan medical assessment and receive focused, Ramadan-specific education on diabetes management. During the fasting period, they are advised to perform regular self-monitoring of blood glucose (SMBG), adhere to an individualized medication adjustment plan, and follow tailored nutritional and exercise guidance to minimize the risk of acute complications.^6^ However, SMBG frequency should be individualized based on treatment regimen, hypoglycemia risk, and clinical setting, in line with the IDF-DAR Practical Guidelines (2021), in older adults with non-insulin-treated, stable T2DM, routine SMBG offers minimal additional clinical benefit. It often imposes undue cost and treatment burden, leading major reviews and guidelines to advise against its routine use in this group.^7^ Epidemiological data from the multiregional CREED cohort indicate that hypoglycemic episodes occurred in 7.1% of fasting patients with T2DM, rising to 9.2% in high-risk participants and 13.8% in very high-risk cohorts.^8^ Similarly, the DAR-MENA prospective observational study among individuals with T2DM reported a confirmed hypoglycemia incidence of 10.4% (0.22 events per participant per month) during Ramadan. Furthermore, patients with DM in KSA report higher rates of hypoglycemic and hyperglycemic episodes.^9^^,^^10^ Despite these risks, a substantial proportion of patients fast: in the EPIDIAR study, 42.8% of participants with T1DM and 78.7% of those with T2DM observed the fast for at least 15 days.^5^ Consequently, structured pre-Ramadan education programs are essential to equip patients, families, and healthcare professionals with the knowledge required for safe fasting.^11^^,^^12^

This study evaluated the level of T2DM management knowledge before and after Ramadan fasting education intervention.

## Materials and Methods

### Study design and population

This study employed a one-group pre-test/post-test design to assess the impact of structured Ramadan fasting education on diabetes-related knowledge. Conducted at Fatmawati General Hospital (Jakarta, Indonesia) from February to April 2023, the intervention was delivered in both online and face-to-face formats. Eligible participants were Muslim adults (aged ≥18 years) with a confirmed diagnosis of T2DM who expressed the intention to fast during Ramadan. Using a consecutive sampling approach, all patients who met these inclusion criteria and were enrolled in the education program were invited to participate. Baseline knowledge was assessed immediately before the educational session (pre-test), and post-intervention knowledge was measured upon completion of the program (post-test), thereby enabling quantification of knowledge gains attributable to the intervention.

### Study hypothesis

The study's evaluative framework was based on the following hypotheses: the null hypothesis (H_0_) indicated that participation in the Ramadan T2DM management education program would result in no change in participants' knowledge scores, whereas the alternative hypothesis (H_1_) claimed that the educational intervention would lead to a positive average increase in knowledge.

### Sample size

The minimum sample size in this study was calculated using the single-proportion formula. A *Zα* value of 1.96 (corresponding to a 5% type I error) and a precision (margin of error) of 0.05 were set. According to the Basic Health Research of Indonesia 2018, the prevalence of DM in Indonesia was 8.5%. Using the formula, a sample size of 96 was obtained.

$$N=\frac{Za2xPxQ}{d2}$$

### Ramadan fasting education program

The Ramadan Fasting Education Program at Fatmawati General Hospital is delivered annually, approximately 4 weeks before the onset of Ramadan. A multidisciplinary team, comprising consultant internists, endocrinologists, diabetes nurse specialists, and clinical dietitians, delivers this program. Instructional modules include a pathophysiological overview of DM under prolonged fasting conditions; detailed training in SMBG techniques; individualized medical nutrition therapy and dietary planning; structured physical activity recommendations; evidence-based adjustment of oral hypoglycemic agents and insulin regimens; and standardized protocols for the prevention, recognition, and management of hypoglycemic events during the fasting period.

### Questionnaire and data collection

Online questionnaires were utilized to assess the knowledge of individuals with T2DM regarding diabetes management during Ramadan. The questionnaire used to evaluate participants' knowledge consisted of questions developed by the research team. The questionnaire was structured into two sections. The first section comprised six questions confirming participants' attendance in topics covered by the program, and the second section included 18 questions focusing on the program content across five key domains: fasting risks for individuals with T2DM, Ramadan nutrition planning, physical activity during fasting, the need to adjust certain medications before fasting, and risk stratification for hypoglycemia and when to break the fast. The program's validity and reliability was assessed using Cronbach's alpha, yielding a score of 0.74; therefore, it can be concluded that the instrument is reliable. Participants completed a pre-test before attending the program and were allowed to complete a post-test 1 month after the program. The questions were identical in both the pre-test and post-test.

### Statistical analyses

Data analyses were conducted using IBM SPSS Statistics version 22 (Armonk, NY, USA). Data normality was tested using Kolmogorov–Smirnov, whereas comparative analysis used the paired *t*-test or Wilcoxon test. Significance was set at p < 0.05 with a 95% confidence interval.

## Results

Of the 116 subjects who met the inclusion criteria, the cohort was predominantly middle-aged and female, with 60.3% between 41 and 60 years old and 62.9% being female. Educational attainment was relatively high, with 45.7% having completed tertiary education. In addition, 46.6% of participants had been living with T2DM for more than 10 years. Regarding glycemic management, most participants (54.3%) were prescribed oral hypoglycemic agents in combination with dietary modification, whereas 36.2% received insulin therapy along with dietary intervention; the remaining participants were managed with other combination regimens (Table 1).

**Table 1:** Baseline characteristics of the study population.

Variables	N	%
Age		
≤40 years	14	12.1
41–60 years	70	60.3
>60 years	32	27.6
Sex		
Male	43	37.1
Female	73	62.9
Education		
Low	23	19.8
Middle	40	34.5
High	53	45.7
Diabetes duration		
<5 years	35	30.2
5–9 years	27	23.3
≥10 years	54	46.6
Therapy		
Oral + diet	63	54.3
Insulin + diet	42	36.2
Insulin + oral + diet	11	9.5
Educational level		
Primary	22	20
Secondary	41	35.3
Tertiary	53	45.7

**Figure 1:**
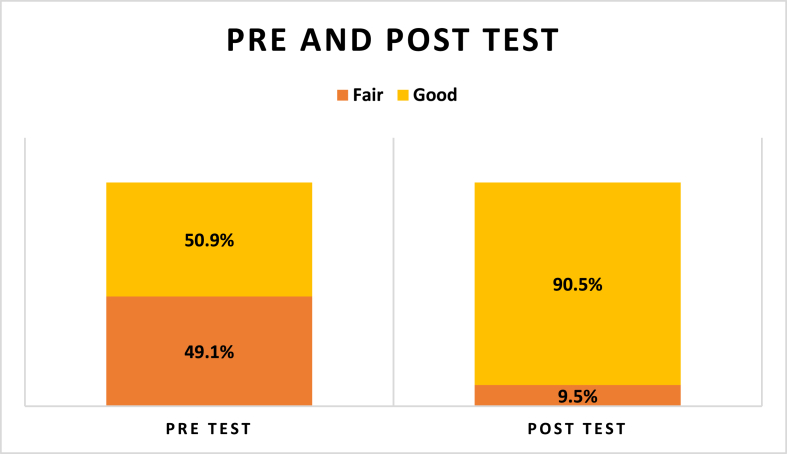
Participant's score pre and post education.

Figure 1 shows that at baseline, 50.9% of participants demonstrated a “good” level of knowledge regarding diabetes management during Ramadan; after the educational intervention, this proportion increased significantly to 90.5%, while the “fair” knowledge category decreased by more than one-third. Change scores were then categorized as positive (knowledge gain), negative (knowledge loss), and unchanged, as shown in Table 2. Specifically, 86 participants (71.6%) showed a positive change, 33 (28.4%) showed a negative change, and 13 (10.8%) showed unchanged. A Kolmogorov–Smirnov test of the change-score distribution indicated significant non-normality (p = 0.001), justifying the use of non-parametric analysis. Consequently, the Wilcoxon signed-rank test confirmed a statistically significant increase in knowledge scores after the intervention (p = 0.001), demonstrating the effectiveness of the Ramadan fasting education program in improving participants’ understanding of diabetes self-management during fasting.

**Table 2:** Proportion of change in study participants’ mean score of knowledge.

Change in mean score	N	%
Positive	83	71.6
Negative	33	28.4
None	13	17.2
Asymp. Sig.	<0.001	

n = number of subjects.

Table 3 presents the proportion of baseline and endline data based on domain questions. The first domain showed that 63.8% of participants correctly recognized that fasting poses risks to individuals with T2DM before education, increasing to 92.2% post-intervention. In the second domain, the majority (87.1%) were already aware of the importance of meal planning before fasting; however, only 37.9% understood the role of complex carbohydrates in sustaining blood sugar levels at baseline, which improved to 63.8% post-education. The third domain showed that the proportion of respondents who correctly stated that individuals with T2DM should not perform intensive exercise while fasting increased from 69.0% to 78.5%. The next domain showed that most participants correctly understood the need to adjust metformin and insulin dosages during fasting and readjust post-Ramadan. However, knowledge regarding the hypoglycemia risk of glibenclamide was initially low (58.6%), but improved to 81.0% after the intervention. High baseline knowledge (average 83.9%) improved to 92.0% after the program. Most participants understood that fasting should be broken due to low or high blood glucose levels or other symptoms, such as dehydration. However, only 67.2% correctly answered the question about the frequency of blood glucose monitoring.

**Table 3:** Proportion baseline and endline based on domains questions.

Domains and Questions	Baseline		Endline	
	True (%)	False (%)	True (%)	False (%)
**Fasting risks for people with diabetes (Domain 1)**				
People with diabetes are at risk when fasting during Ramadan	63.8	36.2	92.2	7.8
Hyperglycemia is one of the risks of fasting during Ramadan for people with diabetes	56.0	44.0	87.1	12.9
I know that I am classified as low, moderate, or high risk if I fast during Ramadan	62.9	37.1	90.5	9.5
If I am at high risk, I am still allowed to fast	50.0	50.0	86.2	13.8
**Ramadan nutrition planning (Domain 2)**				
Nutrition/diet planning for Ramadan fasting should be done before I begin fasting	87.1	12.9	94.8	5.2
Foods containing complex carbohydrates (rice, noodles, vermicelli, bread, sweet potatoes, cassava, potatoes, corn, etc.) cannot maintain blood sugar levels for a longer period, causing people to feel hungry more quickly	37.9	62.1	63.8	36.2
Consuming caffeinated beverages can increase the risk of dehydration	64.7	35.3	84.5	15.5
**Physical activity during fasting (Domain 3)**				
People with diabetes should not exercise while fasting during Ramadan	69.0	31.0	78.5	21.6
Tarawih prayers can be considered part of physical activity during Ramadan fasting	98.3	1.7	91.4	8.6
**The need for adjusting certain medications before fasting (Domain 4)**				
People with diabetes who usually take metformin 3 times a day before fasting need to adjust their metformin dosage during sahur or iftar according to doctor's advice	91.4	8.6	92.2	7.8
The use of glibenclamide during Ramadan fasting can increase the risk of hypoglycemia	58.6	41.4	81.0	19.0
People with diabetes who change their insulin dosage while fasting during Ramadan need to readjust their insulin dosage after the fasting month ends	87.1	12.9	91.4	8.6
**Risk stratification for hypoglycemia and when to break the fast (Domain 5)**				
Checking blood glucose during fasting will invalidate the fast	85.3	14.7	92.2	7.8
Self-monitoring of blood glucose during fasting is done 8 times a day	82.8	17.2	67.2	32.8
I already know the symptoms of hypoglycemia	72.4	27.6	93.1	6.9
If I am fasting, I need to break the fast if my blood glucose level is < 70 or if there are symptoms of hypoglycemia	90.5	9.5	92.2	7.8
If I am elderly, I do not need to perform self-monitoring of blood glucose	87.1	12.9	93.1	6.9
If I am fasting, I need to break the fast if my blood glucose level is > 300, if there are symptoms of dehydration, or if I am ill	84.5	15.5	90.5	9.5

## Discussion

A total of 116 individuals completed our structured Ramadan-focused diabetes education program, of whom 62.9% were women. This female predominance exceeds the 52.4% reported in a previous multicenter study of Muslims with T2DM who fasted during Ramadan, suggesting that site-specific patient demographics and the use of consecutive sampling may have contributed to an overrepresentation of women in our cohort.^13^ Cultural factors and sex differences in health-seeking behaviors may also explain this disparity, as women often engage more proactively with preventive healthcare services. Therefore, when interpreting the generalizability of our findings, it is important to consider potential sampling biases, and future research should use stratified recruitment strategies to ensure balanced sex representation.^14^

After the intervention, their knowledge scores markedly increased (p < 0.001), demonstrating that such an intervention substantially enhances understanding of diabetes management during fasting. At baseline, just over half of the cohort already possessed “good” knowledge, but this rose to 90.5% following the educational sessions. These gains surpass those reported in KSA, where only 4.7% of patients demonstrated high knowledge and 29.2% low knowledge, and in Malaysia, where just 24.2% achieved good knowledge.^15^^,^^16^ Conversely, results from Jordan (53.5% with good knowledge) were similar to our findings.^17^ The improvement in knowledge was correlated with more positive attitudes toward diabetes care, underscoring the value of formal education in empowering patients to manage their condition effectively. Indeed, a Brazilian study also highlighted that diabetes education programs can enhance patients' understanding of disease mechanisms, treatment principles, self-monitoring practices, potential complications, and the role of family support.^18^ Several methodological elements, such as the characteristics of the enrolled population and the specific content or questions assessed, likely contributed to the observed gains in knowledge. Following the educational intervention, approximately 70% of participants demonstrated a statistically significant improvement in their average knowledge scores. At the same time, the share of individuals with only “fair” knowledge decreased by more than one-third. These results demonstrate that a Ramadan-focused educational program can meaningfully enhance participants’ understanding. This conclusion is consistent with Istianah et al., who also found that targeted education significantly improved diabetes knowledge.^19^

While the majority of participants demonstrated improved knowledge after the intervention, approximately 28% exhibited a decline in post-intervention test scores. Such unexpected decreases may reflect cognitive overload, where the volume or complexity of material exceeds learners' working-memory capacity and impairs retention.^20^ Information overload in patient education has been identified as a barrier to effective learning when material surpasses an individual's “absorbing threshold,” leading to confusion and reduced performance.^21^ Likewise, test fatigue, a decline in engagement and concentration due to repeated or lengthy assessments, has been shown to contribute to poor knowledge retention in diabetes education, regardless of patients' baseline literacy levels.^22^ Finally, a misunderstanding of terminology or concepts may persist when patients receive didactic information without a sufficient opportunity for clarification. Methods such as teach-back, which require patients to restate information in their own words, have been recommended to uncover and remediate these gaps.^23^

The educational intervention focused on five key areas: recognizing fasting-related risks in individuals with T2DM, providing guidance on nutritional planning including food and fluid intake, offering recommendations for safe physical activity during Ramadan, making appropriate medication adjustments, and establishing clear criteria for when to break the fast. In the first area, participants’ ability to identify fasting-related hazards significantly improved from 63.8% at baseline to 92.2% after the intervention. However, many high-risk individuals still chose to fast. For the item *“If I am at high risk, I am still allowed to fast,”* 50% of participants answered “yes” at baseline, rising to 86% after education. This likely reflects the influence of cultural and religious beliefs, where decisions are shaped by previous experiences as well as guidance from healthcare providers, religious leaders, and family members.^24^

This paradox illustrates how cultural, spiritual, and psychosocial factors can outweigh medical recommendations. Previous studies, including the EPIDIAR, CREED, and DAR-MENA studies, demonstrated that many individuals with diabetes continue to fast during Ramadan despite being categorized as high risk and being advised otherwise, often due to spiritual, social, or personal reasons.^5^^,^^8^^,^^9^ The increase observed in our study suggests that while knowledge improved, participants framed the information within their own cultural and religious context, exercising autonomy in decision-making. This highlights the persistent gap between understanding and behavioral change, emphasizing the need for collaboration between healthcare providers, religious leaders, and community stakeholders to support safer fasting practices.^33^

In the nutritional area, although 87.1% of participants already recognized the value of structured meal planning before Ramadan, less than 40% initially understood how complex carbohydrates can help maintain normal blood sugar levels; targeted education increased this understanding to 63.8%. These findings support previous research indicating that targeted dietary education is crucial for reducing blood sugar fluctuations and maintaining proper hydration during extended fasting periods.^25^ Ramadan fasting significantly impacts physiological homeostasis by altering sleep patterns, circadian rhythms, fluid and energy balance, and glucose regulation due to the complete abstention from food and drink during daylight hours for an entire lunar month.^6^^,^^26^

In our study, domains related to physical activity and medication management (Domains 3 and 4) demonstrated notable improvements in knowledge. This was reflected by the increased proportion of participants who recognized that individuals with T2DM should avoid intensive exercise, which rose from 69.0% at baseline to 78.5% after the intervention, indicating a better understanding of safe activity practices. Interestingly, the proportion of participants who considered Tarawih prayers as a form of exercise decreased from 98% to 91% after education. This finding may indicate a more nuanced understanding: although Tarawih prayers involve repeated cycles of standing, bowing, and kneeling and can be considered as contributing to physical activity during Ramadan, they may not fully substitute for structured, moderate-intensity exercise recommended for individuals with diabetes. This interpretation is consistent with the IDF-DAR Practical Guidelines and prior reviews, which acknowledge Tarawih as part of daily activity but emphasize the need to maintain regular exercise for optimal diabetes management.^34^^,^^6^ Nevertheless, ongoing education is essential to both alleviate fears of hypoglycemia and encourage engagement in light to moderate exercise, including Tarawih prayers, which can provide cardiovascular and metabolic benefits without substantially increasing the risk of hypoglycemia.^27^ Regarding medication, most participants correctly understood the need to adjust doses of metformin and insulin during Ramadan and to readjust them afterward. Notably, awareness of the risk of hypoglycemia associated with glibenclamide, a sulfonylurea contraindicated in high-risk fasting individuals, improved substantially from 58.6% to 81.0% following the educational program.^6^ These results highlight the effectiveness of the intervention in promoting safer medication practices and emphasize the importance of targeted education in reducing fasting-related complications among individuals with T2DM.

In the final domain, risk stratification and criteria for breaking the fast, participants exhibited a high level of baseline knowledge (mean correct responses 83.9%), which further increased to 92.0% following the educational intervention. Most respondents accurately identified clinical indications for fast termination, including symptomatic hypoglycemia, symptomatic hyperglycemia, and signs of dehydration, underscoring effective transmission of critical safety concepts. Nonetheless, only 67.2% of participants correctly reported the recommended frequency of blood glucose monitoring during Ramadan. We acknowledge the inaccuracy regarding the questionnaire item stating “Self-monitoring of blood glucose during fasting is done 8 times a day” is not evidence-based and may have contributed to confusion among participants. According to the IDF-DAR Practical Guidelines 2021, the frequency of SMBG during Ramadan should not follow a fixed number but instead be tailored to the individual's risk profile, medication regimen, and overall clinical context. Overgeneralized or rigid recommendations can lead to misunderstandings, as evidenced by the increased number of incorrect responses to this item in the post-test. To enhance accuracy and relevance, future education materials should emphasize symptom-driven monitoring and individualized decision-making, encouraging patients to monitor glucose particularly during acute illness, warning signs of hypoglycemia, or when clinically indicated. A clear explanation based on established guidelines is essential to prevent misconceptions and support safe fasting practices.^6^

Individuals with T2DM who observe Ramadan fasting are susceptible to a spectrum of metabolic and vascular complications, including hypoglycemia, hyperglycemia, and dehydration-associated thrombosis, which underscore the importance of risk stratification in this population.^28^^,^^29^ The IDF recommends categorizing individuals into low, moderate, and high-risk strata based on fasting blood glucose levels, with evidence demonstrating a direct relationship between higher risk categories and an increased incidence of hypoglycemic events. Consequently, those classified as high risk are strongly counseled against fasting; nonetheless, a majority of patients with both T1DM and T2DM elect to fast, highlighting the imperative for comprehensive, targeted education to mitigate adverse outcomes.^6^ Comparative studies have shown that patients receiving structured Ramadan-focused instruction engage in safer fasting behaviors, such as making appropriate medication adjustments, being vigilant in self-monitoring, and ceasing the fast promptly, more consistently than their untrained counterparts.^30^^,^^31^ Moreover, effective educational interventions should extend beyond patients to encompass family members and healthcare providers, thereby fostering a supportive environment that reinforces safe fasting practices.^6^^,^^32^

Ramadan-focused educational interventions have been shown to facilitate significant behavioral adaptations among patients with T2DM, enabling them to implement appropriate dietary and activity regimens during the fasting month. These programs reduce the incidence of hypoglycemia and prevent excessive weight gain—outcomes that are directly linked to improved glycemic and metabolic control.^27^ Given these benefits, structured pre-Ramadan education must be offered to all individuals with T2DM who plan to fast. However, the scope of such initiatives should extend beyond patients to include healthcare professionals and key members of their social support network. Clinicians, particularly physicians and diabetes educators, must receive targeted training to reinforce patient responsibility for health and treatment adherence, employing culturally and religiously consonant motivational strategies where appropriate. Initiating education 4–8 weeks before Ramadan allows adequate time for individualized risk stratification, medication adjustment, and rehearsal of self-management behaviors. When delivered effectively, pre-Ramadan programs enhance patients’ knowledge, self-efficacy, and ability to modify lifestyle factors, such as meal planning, blood glucose monitoring, and safe physical activity, thereby minimizing both acute complications and long-term adverse outcomes associated with fasting.^6^^,^^33^

This study highlights the significance of Ramadan-focused education for patients with T2DM, particularly in countries with substantial Muslim populations, and provides a valuable resource for future research. However, several limitations should be noted. First, the single-arm pre–post design without a concurrent control group restricts our ability to attribute knowledge improvements solely to the intervention, as external factors (such as maturation, testing effects, or other educational experiences) might have influenced the outcomes. Second, although our self-created questionnaire demonstrated acceptable internal consistency, it may not encompass the entire range of diabetes-specific knowledge or align with established international tools. Third, the 1-month follow-up period did not allow us to evaluate long-term retention of knowledge or ongoing behavioral changes during Ramadan. Lastly, social desirability bias may have influenced participants’ responses. Since we focused on knowledge scores only, we did not examine actual clinical or behavioral outcomes, which makes the real-world impact of the educational program uncertain.

## Conclusion

The Ramadan fasting education program yielded a marked improvement in diabetes-related knowledge, with structured sessions enhancing participants' understanding across key domains, risk assessment, medication management, and safe fasting practices, thereby helping to minimize adverse events during the month. However, the findings also highlight gaps in certain areas, including guidance on optimal dietary choices, awareness of sulfonylurea-associated hypoglycemia risks, and standardized protocols for self-monitoring of blood glucose. To address these gaps, future curricula should place greater emphasis on culturally and religiously tailored content, ensuring that educational messages resonate with patients’ beliefs and practices, thereby promoting sustained adherence to safe fasting strategies.

## Ethical approval

Confidentiality measures were maintained, and the study proceeded after receiving approval from the Fatmawati General Hospital Research Ethics Committee (Approval No. UM.01.05/VIII.5/115/2023).

## Authors contributions

AAAW Conceptualization, Methodology, Software, Validation, Formal analysis, Investigation, Resources Data Curation, Writing - Original Draft, Project administration, Funding acquisition, Visualization. NHM Software, Formal analysis, Investigation, Resources Data Curation, Visualization, Writing - Original Draft, Project administration, Visualization. JN Conceptualization, Methodology, Software, Validation, Formal analysis, Investigation. MIM Conceptualization, Validation, Formal analysis, Investigation, Resources, Review & Editing Supervision, Funding acquisition. ME Conceptualization, Validation, Formal analysis, Investigation, Resources, Review & Editing Supervision, Funding acquisition. IS Formal analysis, Review & Editing Supervision. IAMK Conceptualization, Review & Editing Supervision, Funding acquisition. All authors have critically reviewed and approved the final draft and are responsible for the content and similarity index of the manuscript.

## Declaration of generative AI and AI-assisted technologies in the writing process

During the preparation of this work, the author(s) used OpenAI ChatGPT to improve readability and language. After using this tool/service, the authors reviewed and edited the content as needed and take full responsibility for the publication's content.

## Source of funding

This research did not receive any specific grant from funding agencies in the public, commercial, or not-for-profit sectors.

## Conflict of interest

The authors have no conflict of interest to declare.
