# Supplementary material for: Improving knowledge on the management of diabetes mellitus during Ramadan
Source: J Taibah Univ Med Sci. 2025 Nov 19;20(6):784–92. doi: 10.1016/j.jtumed.2025.10.005 (PMC12799565; doi:10.1016/j.jtumed.2025.10.005)
Supplement: Multimedia component 1 [file mmc1.docx]

**Supplementary Information**

The questionnaire used for data collection in the study “Knowledge Improvement on Ramadan Diabetes Mellitus Management Among Patients with Type 2 Diabetes Mellitus”.

1. Attendance-Related Questionnaire: Preliminary Questions After Education (Yes/No Questions**)**

These questions are designed to confirm whether participants attended all the educational topics provided by the program.

1. I participated in group education on the topic of Diabetes and Fasting during Ramadan. (Y/N)
2. I participated in group education on the topic of Meal Planning during Ramadan Fasting. (Y/N)
3. I participated in group education on the topic of Exercise during Ramadan Fasting. (Y/N)
4. I participated in group education on the topic of Managing Oral Diabetes Medication during Ramadan Fasting. (Y/N)
5. I participated in group education on the topic of Managing Insulin Injections during Ramadan Fasting. (Y/N)
6. I participated in group education on the topic of Hypoglycemia and Self-Monitoring of Blood Glucose during Ramadan Fasting. (Y/N)

B**.** Content-Related Questionnaire

These questions are given twice: pre and post education (True or False / Yes or No Questions)

1. People with diabetes are at risk when fasting during Ramadan. (T/F)
2. Hyperglycaemia is one of the risks of fasting during Ramadan for people with diabetes. (T/F)
3. I know that I am classified as low, moderate, or high risk if I fast during Ramadan. (Y/N)
4. If I am at high risk, I am still allowed to fast. (T/F)
5. Nutrition/diet planning for Ramadan fasting should be done before I begin fasting. (T/F)
6. Foods containing complex carbohydrates (rice, noodles, vermicelli, bread, sweet potatoes, cassava, potatoes, corn, etc.) cannot maintain blood sugar levels for a longer period, causing people to feel hungry more quickly. (T/F)
7. Consuming caffeinated beverages can increase the risk of dehydration. (T/F)
8. People with diabetes should not exercise while fasting during Ramadan. (T/F)
9. Tarawih prayers can be considered part of physical activity during Ramadan fasting. (T/F)
10. People with diabetes who usually take metformin 3 times a day before fasting need to adjust their metformin dosage during sahur or iftar according to doctor's advice. (T/F)
11. The use of glibenclamide during Ramadan fasting can increase the risk of hypoglycemia. (T/F)
12. People with diabetes who change their insulin dosage while fasting during Ramadan need to readjust their insulin dosage after the fasting month ends. (T/F)
13. Checking blood glucose during fasting will invalidate the fast. (T/F)
14. Self-monitoring of blood glucose during fasting is done 8 times a day. (T/F)
15. I already know the symptoms of hypoglycemia. (Y/N)
16. If I am fasting, I need to break the fast if my blood glucose level is <70 or if there are symptoms of hypoglycemia. (T/F)
17. If I am elderly, I do not need to perform self-monitoring of blood glucose. (T/F)
18. If I am fasting, I need to break the fast if my blood glucose level is >300, if there are symptoms of dehydration, or if I am ill. (T/F)
